# Aortic Perforation and Ascending Dissection Due to Intra-Aortic Balloon Counterpulsation Implantation in Cardiac Surgery

**DOI:** 10.1016/j.jaccas.2022.05.029

**Published:** 2022-08-03

**Authors:** Francisco J. González-Ruiz, Gustavo Rojas-Velasco, Emmanuel A. Lazcano-Diaz, Daniel Manzur-Sandoval, Luis A. Baeza-Herrera, Luis A. Cota-Apodaca, Eduardo R. Bucio-Reta, Angel Ramos-Enriquez, Christian Fernando García-Flores, Guering Eid-Lidt

**Affiliations:** a Department of Cardiovascular Critical Care, National Institute of Cardiology “Dr. Ignacio Chávez,” México City, México; b Department of Interventional Cardiology, National Institute of Cardiology “Dr. Ignacio Chávez”, México City, México

**Keywords:** aortic dissection, aortic perforation, cardiac surgery, endovascular repair, intra-aortic balloon pump, vascular complications, IABP, intra-aortic balloon pump

## Abstract

The intra-aortic balloon pump continues to be a useful ventricular assist device in cardiac surgery. Complications are estimated to be 7% to 40%, significantly high to catastrophic. We describe an aortic injury associated with the use of the device and an interdisciplinary management for the diagnostic and therapeutic approach. (**Level of Difficulty: Intermediate.**)

## Central Illustration

**Figure:**
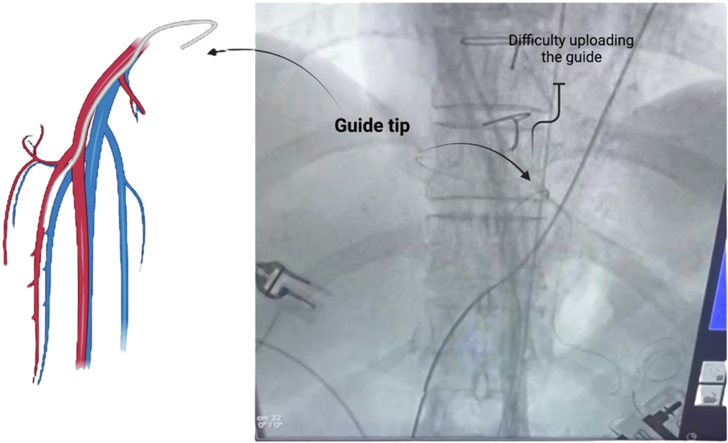

A 63-year-old woman with a history of diabetes, hypertension, and chronic ischemic heart disease was admitted to the emergency department for angina at rest. The initial echocardiogram showed a left ventricle with concentric hypertrophy and mobility alterations in the anterior and anterolateral territory with an ejection fraction of 54%. She underwent coronary angiography, which revealed diffuse disease of the left anterior descending artery with chronic total occlusion. The first diagonal artery was calcified, with chronic total occlusion and Thrombolysis In Myocardial Infarction flow grade 0; the circumflex artery showed diffuse disease in the distal segment, compromising the branch of the obtuse margin; and diffuse disease of the right coronary artery was seen, with a lesion of the posterior descending artery of 70%, and a SYNTAX score of 37.5. She underwent coronary revascularization surgery. During the surgical procedure, left mammary artery revascularization to the left anterior descending artery, anastomosis of the saphenous vein to the first diagonal, and obtuse marginal were performed. During the end of the surgical procedure, disconnection from the extracorporeal circulation circuit was difficult, so placement an intra-aortic balloon counterpulsation device was necessary. Resistance to the insertion was not encountered, and the patient was subsequently transferred to intensive cardiovascular therapy. During admission to the critical care unit, ST-segment elevation was identified in DI, aVL, and V_3_ to V_6_, and significant elevation of high-sensitivity troponin I besides deterioration of ventricular function (ejection fraction 20%). She was transferred to the cardiac catheterization room, where graft occlusion was confirmed.

**Learning Objectives**

• To be able to recognize that device-associated vascular complications carry a high mortality.
• To be able to identify these complications rapidly to decrease mortality.
• To demonstrate that interventional endovascular treatment of type B aortic dissection is feasible with very high success rates.

## Question 1: What Is the Next Step in the Care of This Patient?

**Figure 1:**
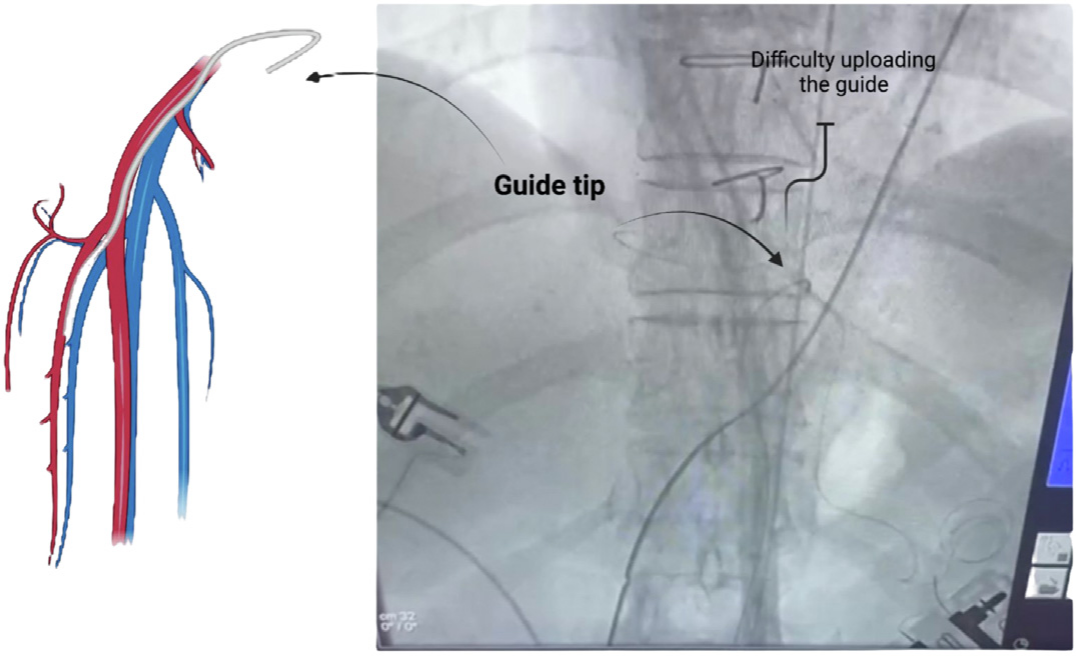
Resistance During Advancement of a 0.0035-Inch Guidewire Then it was decided to perform radial aortography, observing a dissection flap.

The choice of therapeutic strategy depends on the characteristics of each patient. Although conservative medical treatment is chosen, low cardiac output syndrome, hemodynamic instability, and cardiogenic shock are clear indications for reintervention,^1^^,^^2^ whether surgical or percutaneous. The mortality rate in surgical intervention is estimated at 4.6%.^3^ Compared with coronary intervention of native arteries, percutaneous coronary intervention of grafts has shown a significant increase in adverse events, especially in the immediate postoperative period; however, when it is feasible, it offers higher success rates, which are approximately 25% to 34% compared with surgical reintervention.^1^^,^^4^^,^^5^ In the angiographic exploration, percutaneous intervention of the grafts was not possible, and revascularization of the native arteries was not feasible. The arterial approach was femoral, but it was difficult to access the guidewire (Figure 1), so that aortic dissection was suspected. Therefore, the patient underwent computed tomography, which showed ascending aortic dissection and aortic perforation due to the intra-aortic balloon pump (IABP).

## Question 2: What Findings Were Observed by Aortic Tomography?

**Figure 2:**
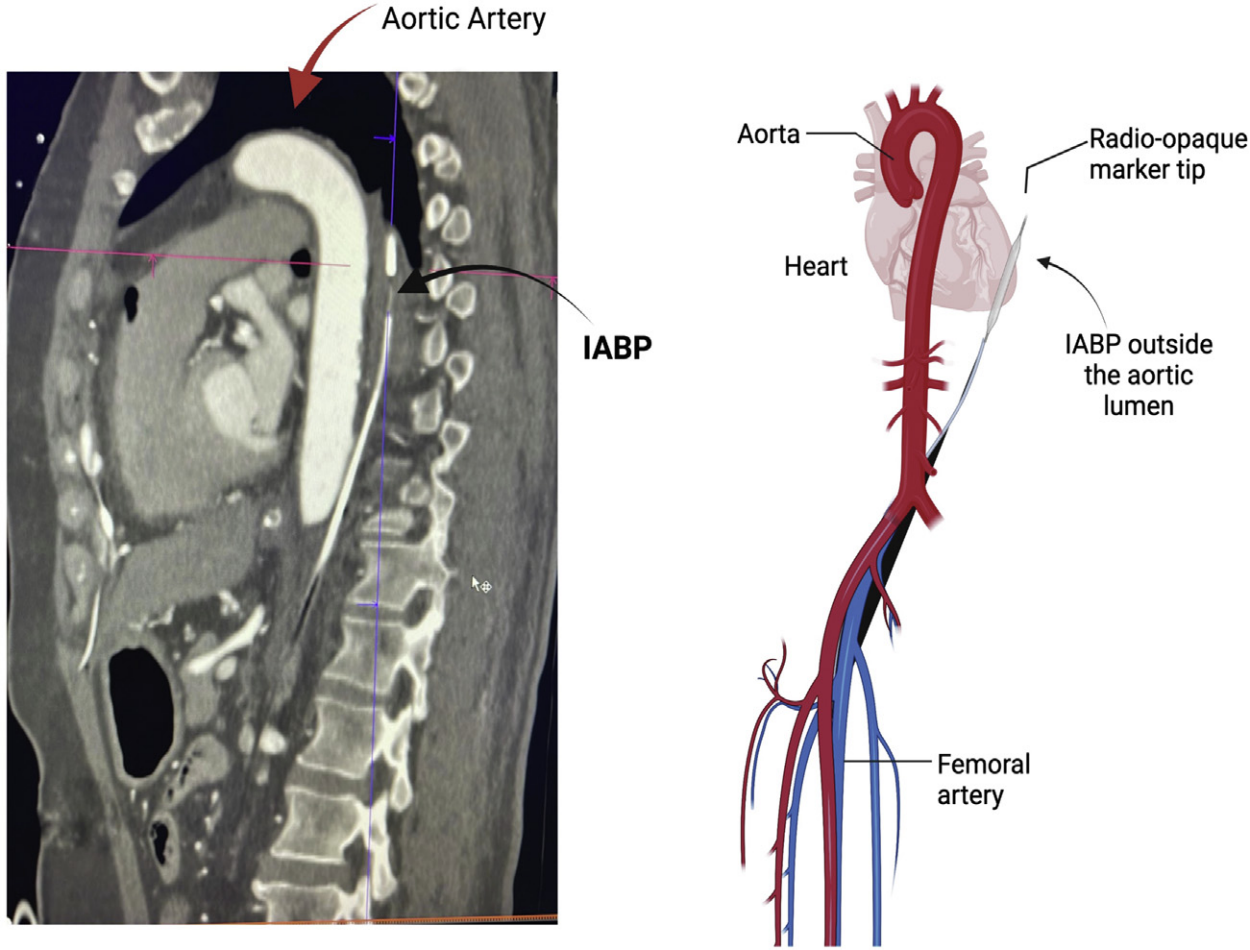
Computed Tomography of Aorta Showing Malposition of IABP Outside Aorta Aortic dissection and perforation were found at the L2 level, compromising the flow of the left renal artery. IABP = intra-aortic balloon pump.

**Figure 3:**
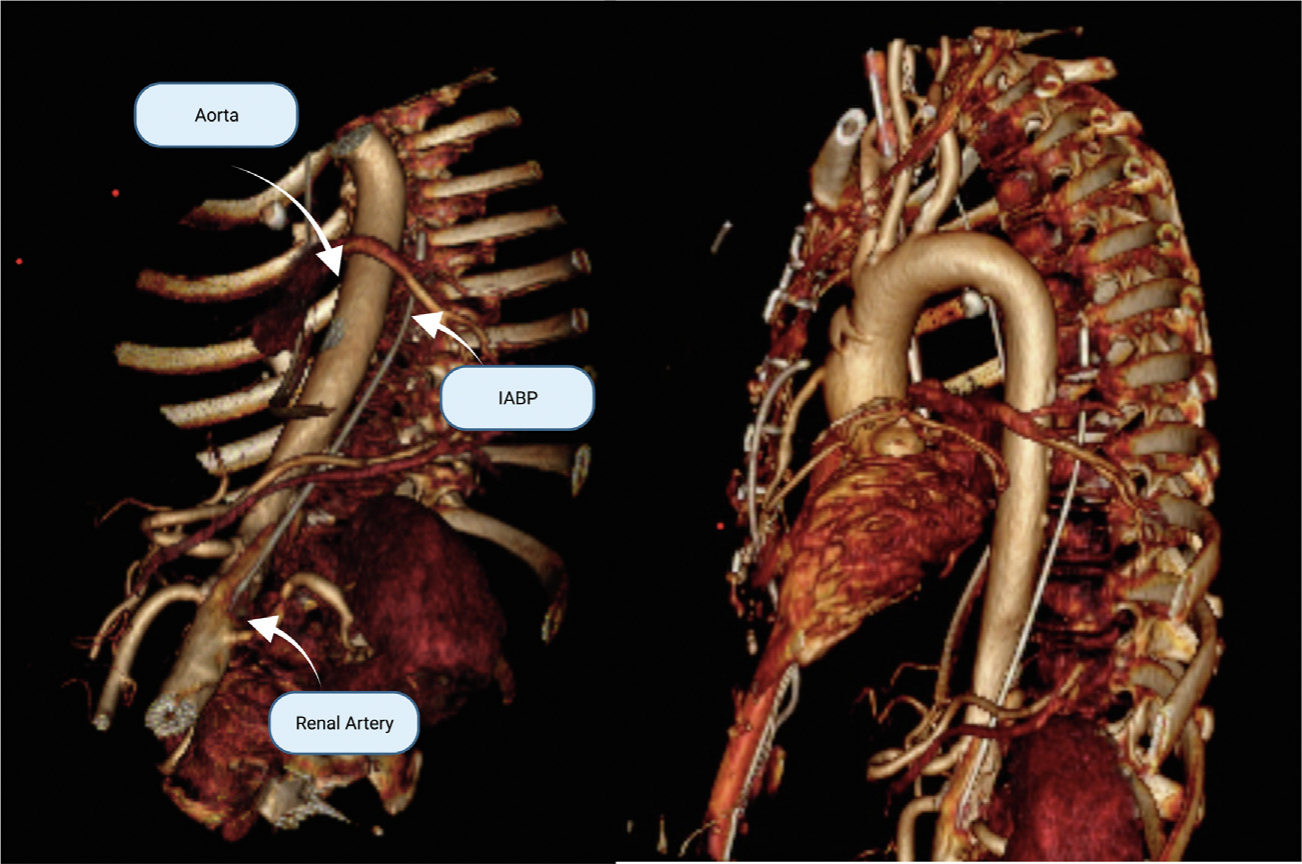
3-Dimensional Reconstruction Reconstruction showing perforation site above the renal artery and its course in the thoracoabdominal cavity. IABP = intra-aortic balloon pump.

A focal dissection flap was observed, beginning at the level of L2 and extending cephalad toward T9, with thrombosis of the proximal portion of the lumen. The IABP was found in the false lumen, perforating the aorta with the distal end at T5-T6 (Figures 2 and 3). A left paravertebral collection with a density of 50 HU was observed, which suggested blood collection.

## Question 3: What Is the Conduct to Follow in the Management of the Aortic Injury?

After an extensive discussion with the multidisciplinary team of critical cardiology, vascular surgery, cardiac surgery, and interventional cardiology, it was determined that the mortality associated with vascular surgery for aortic repair was high, so a collective decision to withdraw the IABP and to pursue endovascular treatment of aortic dissection and perforation would be the correct course of action.

## Question 4: What Does It Consist of, and What Is the Success Rate of Endovascular Treatment in Aortic Dissection and Treatment of Perforation?

**Figure 4:**
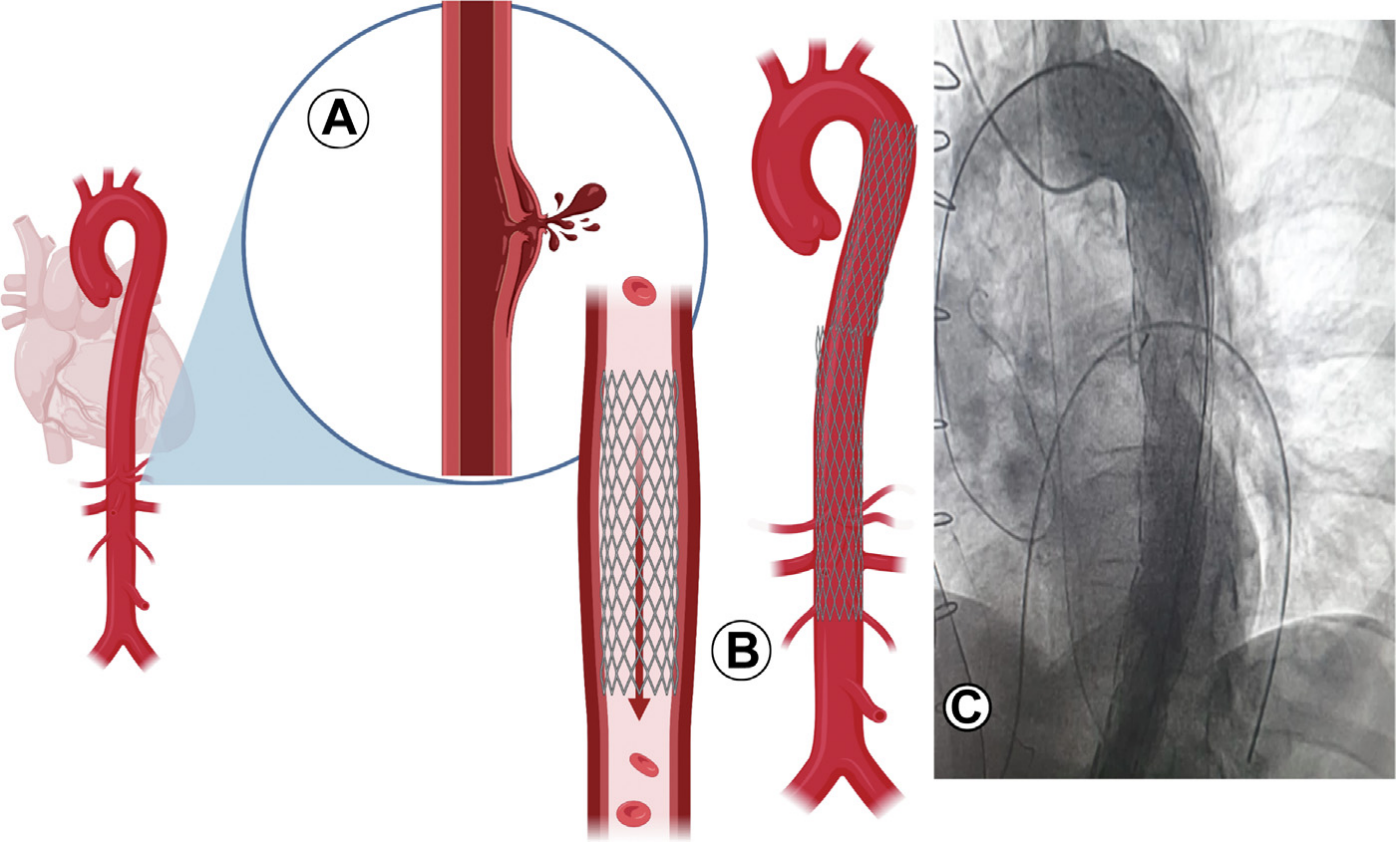
Aortic Perforation **(A)** Endovascular treatment in type B aortic dissection. **(B)** Angiographic projection of endovascular prosthesis distal to the subclavian artery and released in the abdominal aorta **(C)**.

Risk stratification in the treatment of aortic dissection and early identification of this entity allows for appropriate treatment selection. Endovascular treatment is the therapeutic alternative of choice, with low complication rates and high success rates (Figure 4). The fundamental objectives are to prevent rupture of the aorta by controlling its expansion and reversing the inadequate tissue perfusion, preventing multiorgan failure.^6^^,^^7^ The mortality rate associated with this intervention in high-risk patients varies between 10.6% and 18.4%, and it was shown in the INSTEAD (Investigation of Stent Grafts in Aortic Dissection) XL trial^8^^,^^9^ that remodeling of the aorta after 5 years reaches 90.6% and extends to the results of other trials such as the IRAD (International Registry of Acute Aortic Dissection).^10^ From a right radial approach, a pigtail catheter was placed in the aortic arch to perform aortography (Video 1). Through the same vascular access, a hydrophilic guidewire was inserted. Simultaneously, the IABP was removed, and the guidewire was advanced to the femoral region. Without complications, it was possible to place a covered, nonfenestrated aortic stent graft (30 × 200 mm). Control aortography showed adequate expansion of the vascular prosthesis and adequate flow with no evidence of contrast leakage.

## Remainder of Hospital Course

Twenty-four hours after the procedure, the patient had improved hemodynamic stability. It was possible to reduce the dose of vasopressors significantly, and there were no decreases in hemoglobin or findings of tissue hypoperfusion. Despite the success of the percutaneous intervention, revascularization of the grafts and native coronary arteries was not possible. Three weeks later, the patient was discharged from the hospital. The rapid identification of a type V perioperative infarction made it possible to identify the vascular injury secondary to the mechanical assistance device used, which might have gone unnoticed.

## Clinical Perspective

This case demonstrates that endovascular treatment in critically ill patients with acute aortic dissection of the descending aorta is feasible, with high success rates and low mortality. In the same way, early identification and high suspicion of this condition, the interventional cardiologist's experience, and the multidisciplinary team's consensus can dramatically lower mortality in complex cases.

### Acknowledgments

The authors thank all the staff in the Cardiovascular Critical Care Unit of the National Institute of Cardiology.

## Funding Support and Author Disclosures

The authors have reported that they have no relationships relevant to the contents of this paper to disclose.

## References

[1] Turk T. (2016). Early graft failure after coronary artery bypass grafting: diagnosis and treatment. Eur Res J.

[2] Arsenescu C., Gaitan A.E., Statescu C., Tintoiu I., Underwood M., Cook S. (2016). Coronary Graft Failure: State of the Art.

[3] Ghanta R.K., Kaneko T., Gammie J.S. (2013). Evolving trends of reoperative coronary artery bypass grafting: an analysis of the Society of Thoracic Surgeons Adult Cardiac Surgery Database. J Thorac Cardiovasc Surg.

[4] Morrison D.A., Sethi G., Sacks J. (2002). Percutaneous coronary intervention versus repeat bypass surgery for patients with medically refractory myocardial ischemia. J Am Coll Cardiol.

[5] Thielmann M., Massoudy P., Jaeger B.R. (2006). Emergency revascularization with percutaneous coronary intervention, reoperation, or conservative treatment in patients with acute perioperative graft failure following coronary artery bypass surgery. Eur J Cardiothorac Surg.

[6] Nienaber C., Fattori R., Lund G. (1999). Nonsurgical reconstruction of thoracic aortic dissection by stent-grafts placement. N Engl J Med.

[7] Cambria R.P., Crawford R.S., Cho J.S. (2009). A multicenter clinical trial of endovascular stent graft repair of acute catastrophes of the descending thoracic aorta. J Vasc Surg.

[8] Nienaber C., Clough R. (2015). Management of acute aortic dissection. Lancet.

[9] Nienaber C., Kische S., Rousseau H. (2013). Endovascular repair of type B aortic dissection: long-term results of the randomized investigation of stent grafts in aortic dissection trial. Circ Cardiovasc Interv.

[10] Fattori R., Montgomery D., Lovato L. (2013). Survival after endovascular therapy in patients with type B aortic dissection: a report from the International Registry of Acute Aortic Dissection (IRAD). J Am Coll Cardiol Intv.

